# Therapeutic Efficacy, Radiotoxicity and Abscopal Effect of BNCT at the RA-3 Nuclear Reactor Employing Oligo-Fucoidan and Glutamine as Adjuvants in an Ectopic Colon Cancer Model in Rats

**DOI:** 10.3390/life13071538

**Published:** 2023-07-11

**Authors:** Debora N. Frydryk Benitez, Mónica A. Palmieri, Yanina V. Langle, Andrea Monti Hughes, Emiliano C. C. Pozzi, Silvia I. Thorp, Marcela A. Garabalino, Paula Curotto, Paula S. Ramos, María L. Paparella, Lucas Polti, Ana Eiján, Amanda E. Schwint, Verónica A. Trivillin

**Affiliations:** 1 Comisión Nacional de Energía Atómica (CNEA), Av. General Paz 1499, San Martin, Buenos Aires C1650KNA, Argentina; debora_nbh@hotmail.com (D.N.F.B.); andre.mh@gmail.com (A.M.H.); eccpozzi@gmail.com (E.C.C.P.); silviathorp@cnea.gob.ar (S.I.T.); marcegarabalino@gmail.com (M.A.G.); paulacurotto@gmail.com (P.C.); paula.s.ramos@outlook.com (P.S.R.); mandyschwint@gmail.com (A.E.S.); 2 Departamento de Biodiversidad y Biología Experimental, Facultad de Ciencias Exactas y Naturales, Universidad de Buenos Aires (UBA), Av. Int. Güiraldes 2160, 4 Piso, Pab. II, Ciudad Autónoma de Buenos Aires C1428EGA, Argentina; monicaalepalmieri@gmail.com; 3 Facultad de Medicina, Instituto de Oncología Ángel H. Roffo (IOAHR), Universidad de Buenos Aires, Av. S. Martín 5481, Área de Investigación, Ciudad Autónoma de Buenos Aires C1417DTB, Argentina; yaninalangle@yahoo.com.ar (Y.V.L.); anamariaeijan@gmail.com (A.E.); 4 Consejo Nacional de Investigaciones Científicas y Técnicas (CONICET), Ciudad Autónoma de Buenos Aires C1425FQB, Argentina; 5 Facultad Odontología, Universidad de Buenos Aires (UBA), M.T. de Alvear 2142, Ciudad Autónoma de Buenos Aires C1122AAH, Argentina; mluisapaparella@gmail.com (M.L.P.); lucasfabianpolti@gmail.com (L.P.)

**Keywords:** BNCT, RA-3 nuclear reactor, Glutamine, Oligo-Fucoidan, radiotoxicity, abscopal effect, BPA and GB-10, biodistribution, lymph nodes, systemic immune response

## Abstract

Boron neutron capture therapy (BNCT) is based on the preferential uptake of ^10^B compounds by tumors, followed by neutron irradiation. The aim of this study was to assess, in an ectopic colon cancer model, the therapeutic efficacy, radiotoxicity, abscopal effect and systemic immune response associated with (BPA/Borophenylalanine+GB-10/Decahydrodecaborate)-BNCT (Comb-BNCT) alone or in combination with Oligo-Fucoidan (O-Fuco) or Glutamine (GLN), compared to the “standard” BPA-BNCT protocol usually employed in clinical trials. All treatments were carried out at the RA-3 nuclear reactor. Boron biodistribution studies showed therapeutic values above 20 ppm ^10^B in tumors. At 7 weeks post-treatment, the ratio of tumor volume post-/pre-BNCT was significantly smaller for all BNCT groups vs. SHAM (*p* < 0.05). The parameter “incidence of tumors that underwent a reduction to ≤50% of initial tumor volume” exhibited values of 62% for Comb-BNCT alone, 82% for Comb-BNCT+GLN, 73% for Comb-BNCT+O-Fuco and only 30% for BPA-BNCT. For BPA-BNCT, the incidence of severe dermatitis was 100%, whereas it was significantly below 70% (*p* ≤ 0.05) for Comb-BNCT, Comb-BNCT+O-Fuco and Comb-BNCT+GLN. Considering tumors outside the treatment area, 77% of Comb-BNCT animals had a tumor volume lower than 50 mm^3^ vs. 30% for SHAM (*p* ≤ 0.005), suggesting an abscopal effect of Comb-BNCT. Inhibition of metastatic spread to lymph nodes was observed in all Comb-BNCT groups. Considering systemic aspects, CD8^+^ was elevated for Comb-BNCT+GLN vs. SHAM (*p* ≤ 0.01), and NK was elevated for Comb-BNCT vs. SHAM (*p* ≤ 0.05). Comb-BNCT improved therapeutic efficacy and reduced radiotoxicity compared to BPA-BNCT and induced an immune response and an abscopal effect.

## 1. Introduction

Boron neutron capture therapy (BNCT) is based on the preferential uptake of ^10^B compounds by tumors, followed by neutron irradiation. A large amount of research and clinical studies of BNCT have been carried out using mainly research reactors as neutron sources [1]. However, the transition to a technology involving accelerators as neutron sources for BNCT is underway [2]. Numerous clinical studies were carried out in countries such as Japan, Taiwan, the United States, Finland, Italy and Argentina, among others, beginning with pathologies that are refractory to standard treatments such as glioblastoma multiforme and melanoma and expanding to head and neck tumors and liver and lung metastases, among others. These studies began employing nuclear reactors as the neutron source. Currently, with the development of neutron beams based on accelerators, which can be installed in a hospital environment, we can expect (and hope for) an increase in the number of clinical trials worldwide and the inclusion of new potential tumor targets [1].

Despite extensive research on boron compounds, only BPA (borophenylalanine) and BSH (sodium borocaptate) have been applied in human clinical studies [3]. GB-10 (sodium decahydrodecaborate) has been used for a pharmacokinetic study in patients with glioblastoma multiforme [4]. A strategy that is being evaluated in the BNCT community to improve tumor boron targeting homogeneity and therapeutic efficacy is to combine the administration of ^10^B compounds with different uptake mechanisms [5]. BPA is a boronated amino acid that is conjugated with fructose to improve its solubility. It is incorporated into the cell by an active mechanism through the LAT 1 transporters and, to a lesser extent, LAT 2 [1]. Tumors overexpress LAT1, while LAT 2 is found in all tissues, generating differential accumulation. GB-10 is a diffusive compound, which is distributed homogeneously, reaching quiescent cell populations. Although GB-10 does not selectively incorporate into tumors, when used in BNCT, selective tumor damage occurs through a selective effect on aberrant tumor blood vessels [6]. Radiodermatitis is an expected event in radiation treatment. However, this may limit the dose that can be applied to tumors and therefore the efficacy of the treatment. Oligo-Fucoidan (O-Fuco), purified from a brown seaweed extract *Laminaria japonica*, has anti-inflammatory and anticancer activities [7]. In cancer patients, the use of fucoidans was shown to reduce inflammation [8]. Glutamine (GLN) was shown to be capable of reducing dermatitis induced by radio/chemotherapy in patients and of inhibiting tumor development in a model of oral cancer [9,10]. When primary tumors are treated with radiation therapies, immunogenic cell death is induced, triggering a cytotoxic immune response against the primary tumor and its metastases [11]. Ionizing radiation is the energy released by unstable nucleus in the way of photons and particles. It induces immunogenic cancer cell death and modulates the presentation of tumor antigens. The changes elicited in the microenvironment within the irradiated field are an important feature in the overall effect. The direct effect of radiation therapy is detrimental to the very sensitive tumor-infiltrating lymphocytes. These effects lead to a temporary selective ablation of immune cells within the irradiated target. Cytotoxic T lymphocytes and antitumor NK cells are depleted, in addition to Tregs that are responsible for suppressing local antitumor immunity. The relative importance of the effect of radiotherapy in these populations is being studied, but it is evident that the detrimental effects of this physical aggression are detected by the immune system not only locally but also systemically [11]. The “Abscopal” effect (ab-scopus, far from the target) refers to the inhibitory action of radiotherapy on tumor growth out-of-field, i.e., at a site distant from the irradiation area. This effect is mediated by radiation-induced immune responses and was originally described by Mole [12]. BNCT was first shown to exert an abscopal effect in an experimental model by Trivillin et al. [13]. Further studies demonstrated the capability of BNCT to induce immunomodulatory effects and contribute to tumor inhibition [14,15]. The aim of this study, employing an ectopic colon cancer model in BDIX rats, was to evaluate the therapeutic efficacy, radiotoxicity and abscopal effect of (BPA+GB-10/Decahydrodecaborate)-BNCT (Comb-BNCT) alone or in combination with O-Fuco or GLN, compared to the “standard” BPA-BNCT protocol usually employed in clinical trials [3]. Likewise, the presence of metastases in the draining inguinal lymph nodes was evaluated. In addition, in the case of the BPA-BNCT group and the Comb-BNCT group alone or in combination with O-Fuco or GLN, the systemic immune response expressed in the spleen was assessed.

## 2. Materials and Methods

### 2.1. Experimental Model

**Figure 1:**
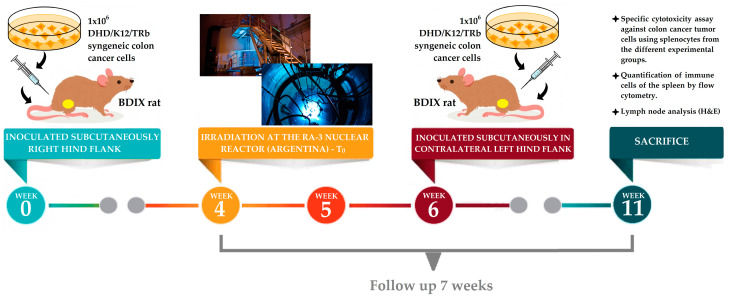
Summary of the experimental steps for BNCT.

An immunocompetent animal model was employed to allow us to analyze not only the local effect of BNCT but also the immune and abscopal effects. BDIX rats were injected subcutaneously (sc) in the right hind flank, under ketamine (36.5 mg/kg bw)-xylazine (5.4 mg/kg bw) anesthesia, with 1 × 10^6^ DHD/K12/TRb syngeneic colon cancer cells (ECACC, Porton Down, Salisbury, UK) in 100 µL of F-10-DMEM culture medium (GIBCO) using a syringe with a 27-gauge needle. Three weeks post-inoculation, when the animals developed subcutaneous measurable, vascularized tumor nodules, they were employed in boron biodistribution and BNCT studies (see Figure 1) [13]. 

Animal experiments were carried out in accordance with the guidelines of the National Institute of Health in the USA regarding the care and use of animals for experimental procedures. Experiments were designed based on the 3Rs (replacement, refinement, and reduction principles), evaluated, and approved by the National Atomic Energy Commission Animal Care and Use Committee (CICUAL-CNEA no. 04/2021). Drinking water and a standard diet (Cooperación-Asociación de Cooperativas Argentina C.L., San Nicolas, Buenos Aires, Argentina) were supplied ad libitum. The room temperature was about 24 °C with a 12/12 h light/dark cycle. Cage changing was performed three times per week. The animals were weighed weekly and their general status was checked periodically. If any type of pain or discomfort was recorded, Tramadol (6 mg/Kg/day, intraperitoneal (ip)) was applied as an analgesic.

### 2.2. Biodistribution Studies

Biodistribution studies are performed to estimate the boron concentration in tumor, blood and clinically relevant normal tissues. These boron concentration values are used to calculate the boron component of the irradiation dose for the different tissues/organs and, in turn, perform dose calculations for BNCT. BPA+GB-10 biodistribution studies were performed in a group of rats bearing one or two tumor nodules each. In some cases, tumors were induced as described above on both legs to obtain a larger amount of sample. BPA (L-enantiomer, >98% ^10^B-enriched; Boron Biologicals, Inc., Raleigh, NC, USA) was complexed with fructose at a ratio of 1:1 M to make it more soluble. The pH was adjusted to 9.5–10 with NaOH, the mixture was stirred until all solids had dissolved, and then the pH was adjusted back to 7.4 with HCl. The solution was passed through a 0.22 nm sterilizing filter (Nalge Company, Rochester, NY, USA) for use. GB-10 (Katchem Ltd., Praha1, Czech Republic, CAS #666747-96-2, >99% ^10^B-enriched) was started from a stock solution at a concentration of 100 mg ^10^B/mL. For administration, a 1:10 dilution with distilled water of the stock solution was made to reach a final concentration of 10 mg ^10^B/mL. BPA 31 mg ^10^B/kg bw plus GB-10 34 mg ^10^B/kg bw were administered intravenously (iv), one after the other, in the jugular vein, surgically exposed under anesthesia. Samples of blood, tumor and skin were taken 3 h post-administration of BPA+GB-10 and processed as previously described for gross boron measurement by using inductively coupled plasma optical emission spectrometry (ICP-OES axial, Agilent 5100) [16]. Briefly, 20–50 mg tumor and normal tissue samples and whole blood samples of approximately 300 mg were taken. Digestions were performed using 0.25 mL of a 1:1 H_2_SO_4_:HNO_3_ solution for tumor samples and normal tissues and 1.25 mL for blood samples at 100 °C for 1 h in a thermal water bath (Vicking Model Dubnoff). Upon reaching room temperature, 0.20 mL of an aqueous solution of Y 0.5 ppm + Sr 25 ppm (internal standards) and 0.55 mL of an aqueous solution of Triton 5% *v/v* were added to the tumor samples and normal tissues. For the blood samples, 1 mL of Y 0.5 ppm + Sr 25 ppm and 2.75 mL of Triton 5% *v/v* were used. In the same way, sample blanks were prepared using the same solutions. To calibrate the equipment, a boric acid standard solution was used.

### 2.3. In Vivo BNCT

**Figure 2:**
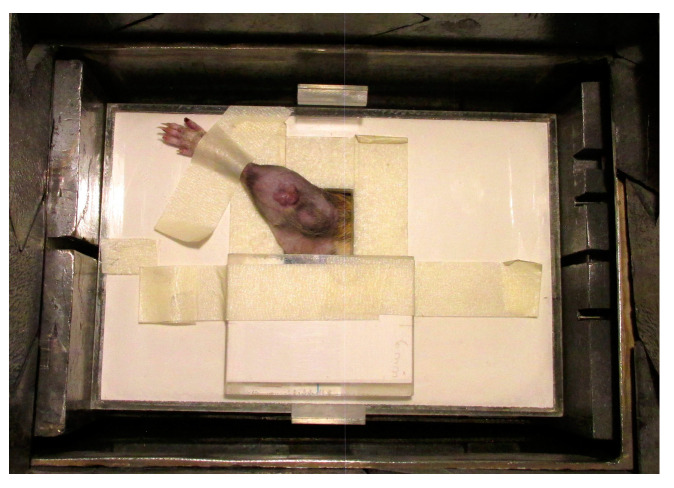
A lithium carbonate thermal neutron shield (enriched to 95% in lithium-6). The tumor-bearing leg is exposed while the shielding protects the rest of the body from the thermal neutron flux.

The tumor-bearing legs were treated locally with BNCT at the RA-3 Nuclear Reactor. A lithium carbonate thermal neutron shield (enriched to 95% in lithium-6), designed and fabricated ad hoc, was used to protect the body of the animal from the thermal neutron flux while exposing the leg through a collimated aperture (Figure 2). The thermal neutron fluence at the irradiation position was 4.2 × 10^12^ n cm^−2^ for all protocols.

This system was positioned in a nearly isotropic neutron field while the reactor was in normal operation. Before each irradiation, a self-powered neutron detector (SPND) was used to perform neutron flux measurements at a monitoring position in order to calculate the exposure time to achieve the prescribed dose [17]. The dosimetric calculations were made using dosimetric data from the RA-3 facility reported by Farías [18]. In Table 1, we show the absorbed doses of the different radiation components, the total absorbed dose of BNCT and the boron concentration data used for dose calculations [13]. The irradiation time to reach the prescribed dose, depending on the neutron flux on the day of irradiation, varied between 18 and 25 min. The thermal neutron fluence at the irradiation position was 4.2 × 10^12^ n cm^−2^, whereas the thermal neutron fluence at all locations within the shield was at least 20 times lower.

### 2.4. Experimental Groups

BDIX rats were assigned at random to each of the following experimental groups.

- **Comb-BNCT**: BPA 31 mg ^10^B/kg bw plus GB-10 34 mg ^10^B/kg bw, iv. n = 13 animals.
- **Comb-BNCT+O-Fuco:** same as (a) plus O-Fuco (200 mg/mL) 5 times a week for 7 weeks, joint oral and topical administration. n = 11 animals.
- **Comb-BNCT+GLN:** same as (a) plus GLN (40 mg/mL) once a week for 7 weeks, with wet compresses. n = 11 animals.
- **BPA-BNCT:** BPA 46.5 mg ^10^B /kg bw, iv. n = 20 animals [14].
- **SHAM:** tumor-bearing rats, same manipulation, no treatment. n = 30 animals.
- **Beam only (BO):** tumor-bearing rats exposed to the same neutron fluence as the BNCT groups, without boron compound administration. n = 10 animals [14].

Two weeks post-irradiation, the same colon cancer cells were injected into the non-irradiated contralateral left hind flank in all rats. We evaluated the abscopal effect in this tumor in the contralateral left leg.

### 2.5. Follow-Up

Weekly, starting from the BNCT treatment (T_0_), and for 7 weeks, measurements of the volume of the tumor nodules (in both legs) were made with a caliper. Tumor volume was determined by measuring the 3 largest orthogonal diameters and expressing the volume in cubic millimeters as A × B × C in accordance with previous studies [14]. The potential inhibitory effect on tumor development in the left leg induced by treatment in the right leg was taken as an indicator of the abscopal effect. The end-point to evaluate tumor growth in the left leg for each group was the incidence of animals with a tumor volume ≤50 mm^3^. Body weight, clinical signs and radiotoxicity on exposed skin were also evaluated.

The percentage of animals with metastatic spread to tumor-draining lymph nodes in the irradiated right leg was analyzed by H&E.

To assess the therapeutic efficacy of BNCT, the post/pre ratio of tumor volume was calculated. Post is the tumor volume at week 7 post-BNCT, and pre is the tumor volume at week 0, prior to treatment. The number of BDIX rats that presented tumors that reduced their volume by at least 50% (post/pre ≤ 0.5) with respect to the initial tumor volume (at the beginning of the treatment) for each experimental group was used to calculate the index % ITVRred ≤ 50%.

**Figure 3:**
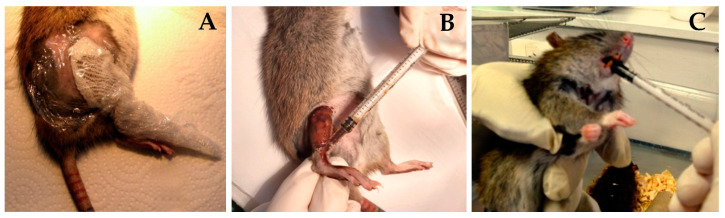
(**A**) A compress moistened with GLN was placed on the leg treated with BNCT. (**B**) O-Fuco was applied topically on the leg treated with BNCT. The leg was then loosely wrapped in film (**C**) Oral administration of O-Fuco by introducing a syringe without a needle on the left side of the mouth of the rats treated by topical administration of O-Fuco.

Once a week from the day of BNCT, when the animals were anesthetized to measure tumor volume, GLN was applied with compresses moistened with GLN diluted in MiliQ water (40 mg/mL) on the treated area. The compresses were wrapped in film as seen in Figure 3A and kept in place until the animal woke up (approximately 30 min).

The O-Fuco was applied topically by painting the treated area, that is, the flank and sole of the animal’s right hind paw (see Figure 3B), and administered orally with a syringe without a needle (see Figure 3C). Thus, O-Fuco was administered at a total dose of 0.4 mL, 0.2 mL by each route, 5 days a week during the 7 weeks of follow-up, starting 1 day before irradiation. 

**Figure 4:**
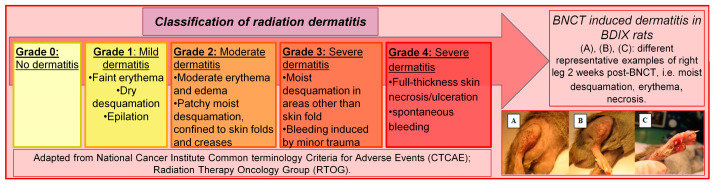
Radiodermatitis scale of the US National Cancer Institute.

The degree of dermatitis of the rats was determined once a week and classified according to the radiodermatitis scale of the US National Cancer Institute as detailed in Figure 4 [19].

### 2.6. Sacrifice

All animals were sacrificed 7 weeks after treatment with an overdose of anesthesia (ketamine (70 mg/kg bw, ip)-xylazine (10.8 mg/kg bw, ip)) and by generating a pneumothorax. At that time, the tumor, skin of the exposed area and inguinal lymph node were removed for histopathological studies. Likewise, the spleen was removed under sterile conditions for cytotoxicity assays and flow cytometry measurement of immune cells.

### 2.7. Cytotoxicity Assay against DHD/K12/TRb Cells

The spleen was removed under sterile conditions, and the splenocytes were obtained by mechanical separation. Splenocytes were stimulated for 4 days with DHD/K12/TRb cells, which were previously inactivated with mitomycin C (100 μg/mL). Splenocytes were then harvested and seeded on a monolayer of live DHD/K12/TRb cells (ratio 10:1 splenocytes:tumor cells). Cytotoxic activity was assessed by MTS (Promega) as absorbance 492/630 nm (Ab). The % cytotoxicity was calculated as: 1 − [Ab(DHD/K12/TRb cells + splenocytes) − Ab(splenocytes)] × 100/Ab(DHD/K12/TRb).

### 2.8. Flow Cytometry

Spleens were mechanically disaggregated using surgical forceps. Spleen cells were harvested. Erythrocytes were lysed with NH4Cl 0.75%, Tris 0.2%, pH 7.2. 1 × 10^6^ splenocytes were centrifuged at 300 g for 7 min and resuspended in 100 μL of PBS + 2% FBS to be labeled with 1 μL CD8 (no. 200609); 2.25 μL CD3 (no. 201412); 0.5 μL CD161 (no. 205608); 1.25 μL CD4 (no. 201509); and 1.25 μL CD25 (no. 202105) from Biolegend for 30 min at 4 °C in darkness. Then, cells were centrifuged at 300× *g* for 7 min and resuspended in 200 μL of 1% formalin in PBS to be analyzed by flow cytometry. For FOXP3 labeling in Treg, after CD4 and CD25 labeling, cells were centrifuged at 300× *g* for 7 min and resuspended in permeabilization buffer (PBS + 0.05% Tween 20 + 2% FBS) for 20 min at 37 °C. In the same buffer, 4.5 μL of FOXP3 (53-4774-42 Thermo Fisher) were added for 30 min at 4 °C in darkness. Then, cells were centrifuged at 300× *g* for 7 min and resuspended in 200 μL of 1% formalin in PBS to be analyzed by flow cytometry. Accuri C6 Plus flow cytometer was used, collecting 50,000 events per sample. Data were analyzed with the Accuri C6 Plus cytometer analysis package.

### 2.9. Statistical Analyses

The local effect at seven weeks, cytotoxicity and systemic evaluation of immune cells were assessed with non-parametric tests such as the Kruskal–Wallis test with Dunn’s comparisons. The local effect over time was evaluated with a two-factor ANOVA of repeated measures with Bonferroni multiple comparisons. The results of radiodermatitis and abscopal effect were compared using Fisher’s test. The database was analyzed with the GraphPad Prism 5.04 software. *p* ≤ 0.05 was considered statistically significant.

## 3. Results

Boron biodistribution studies showed therapeutic values above 20 ppm ^10^B in tumors, as can be seen in Table 2. However, similar values for tumors, surrounding skin and blood were observed for BPA+GB-10. The values were higher than for BPA alone.

The tumor/blood boron concentration ratio is used to indirectly and retrospectively estimate tumor boron values by taking a pre-irradiation blood sample and measuring boron concentration.

The tumor/surrounding skin ratio (T/SS ratio) around 1, both for BPA+GB-10 and for BPA alone, indicates the homogeneous distribution in these tissues. This could also explain the skin radiotoxicity. However, as seen below, animals treated with Comb-BNCT have lower radiotoxicity than those treated with BPA-BNCT alone, despite having higher skin boron concentrations.

**Figure 5:**
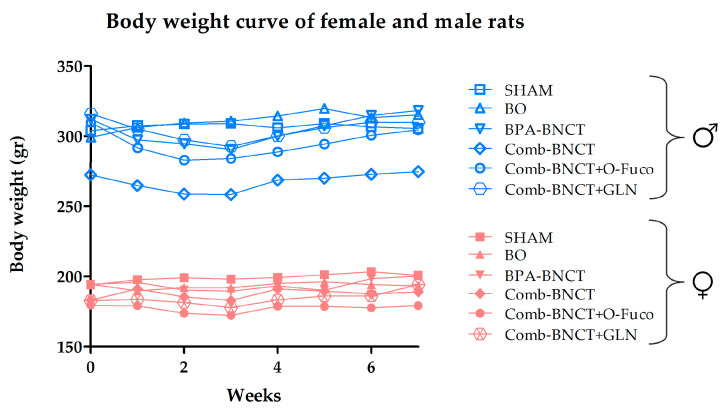
Body weight curve over time of follow-up.

The body weight curve over time (Figure 5) was constructed for males and females since their means are different. No significant differences were observed over time or between groups.

**Figure 6:**
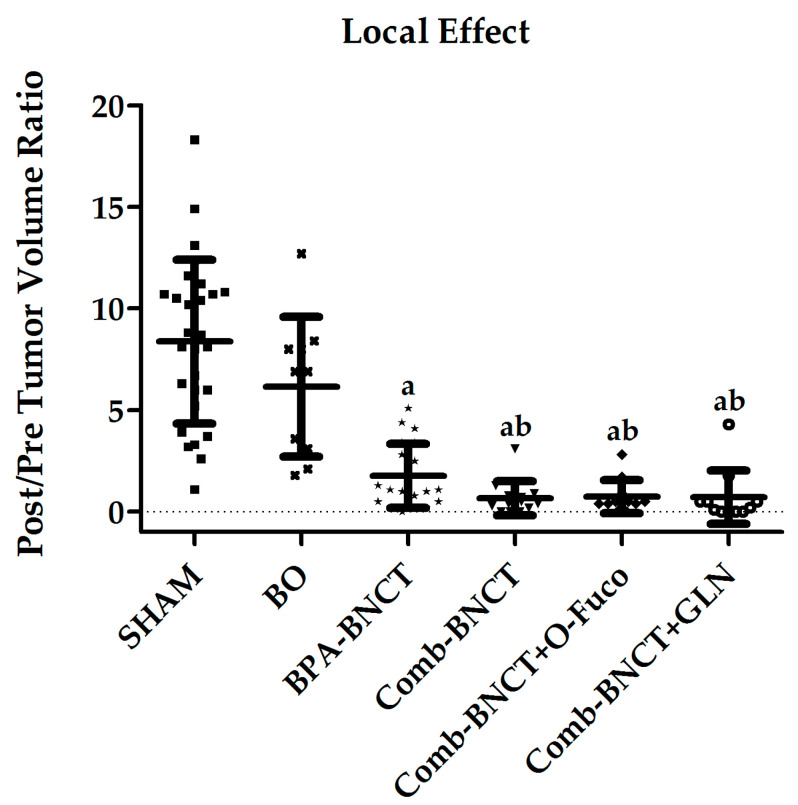
Post/pre-treatment tumor volume ratio (Mean ± SD (N)) vs. experimental group. a = *p* < 0.05 vs. SHAM, b = *p* < 0.05 vs. BO.

The post-/pre-BNCT ratio of tumor volume at 7 weeks post-treatment in the right irradiated leg (Figure 6) was significantly lower for all the groups treated with BNCT vs. SHAM (*p* < 0.05).

**Figure 7:**
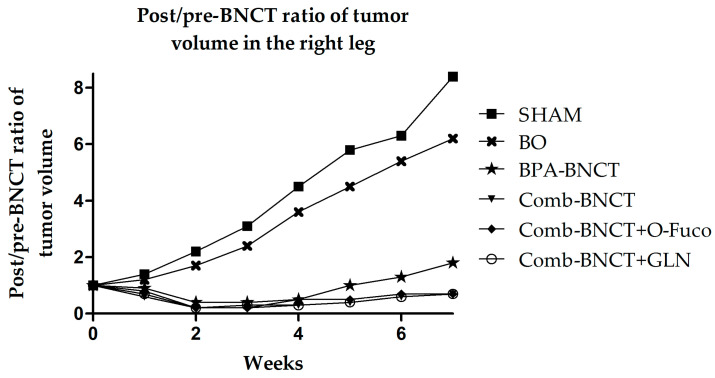
The time progression of the post-/pre-treatment tumor volume ratio in the right leg from 0 to 7 weeks is depicted. To enhance image clarity, the error bars representing the standard deviation have been excluded.

Figure 7 shows the post-/pre-treatment tumor volume ratio throughout the follow-up period. The analysis of variance (ANOVA) revealed significant interactions between groups and week (*p* < 0.0001). Significant differences were observed between the BNCT vs. SHAM groups after 2 weeks (*p* < 0.05), and in subsequent weeks, these differences enhanced (*p* < 0.0001). Likewise, significant differences were observed between all BNCT vs. BO groups from the third week (*p* < 0.05).

**Figure 8:**
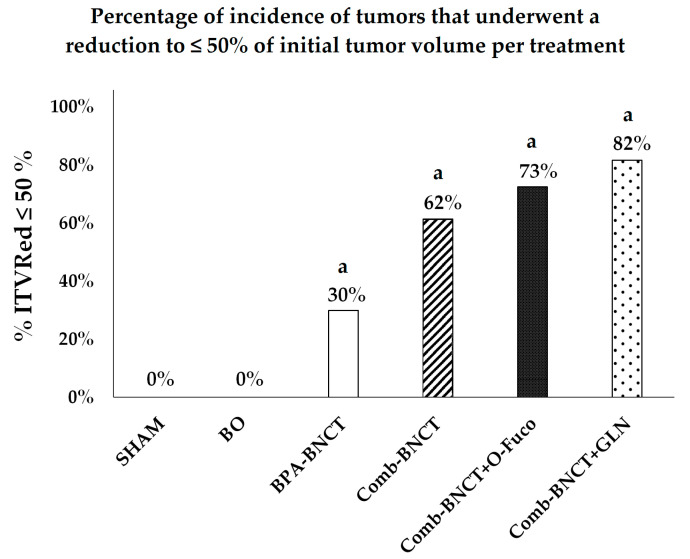
Incidence of tumors that underwent a reduction to ≤ 50% of initial tumor volume (% ITVRed ≤ 50%) vs. experimental group. a = *p* < 0.0001 vs. SHAM.

Using the end-point “incidence of tumors that underwent a reduction to ≤50% of initial tumor volume” (ITVRed ≤ 50%) to further assess the therapeutic response, results were 62% for Comb-BNCT alone, 82% for Comb-BNCT+GLN, 73% for Comb-BNCT+O-Fuco and only 30% for BPA-BNCT (Figure 8).

When we evaluated the metastatic spread to the tumor-draining lymph node in the right leg, we observed inhibition in all groups treated with Comb-BNCT (Table 3). This inhibition was statistically significant for Comb-BNCT vs. SHAM (*p* = 0.0022); Comb-BNCT+GLN vs. SHAM (*p* = 0.0007); and Comb-BNCT+O-Fuco vs. SHAM (*p* = 0.0001). We can see that administration of an adjuvant such as O-Fuco or GLN enhanced the inhibition of metastatic spread to the lymph node. However, these differences did not reach statistical significance. No statistically significant differences were observed between BPA-BNCT vs. SHAM. Likewise, we did not find any differences between BPA-BNCT vs. Comb-BNCT. However, the differences between BPA-BNCT vs. Comb-BNCT+O-Fuco (*p* = 0.016) and BPA-BNCT vs. Comb-BNCT+GLN (*p* = 0.0498) were statistically significant. This would suggest that the application of adjuvants such as O-Fuco and GLN would enhance the inhibition of metastatic dissemination.

**Figure 9:**
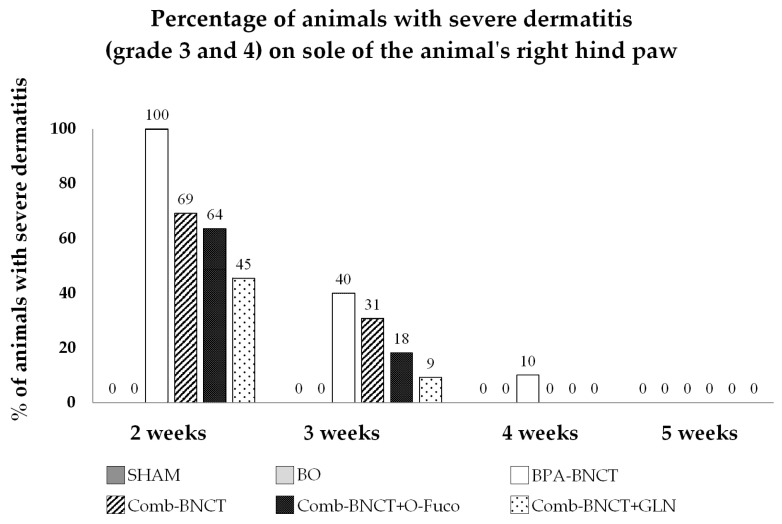
Percentage of animals with severe dermatitis (grade 3 and 4) on sole of the animal’s right hind paw vs. number of weeks.

The incidence of severe dermatitis at two weeks (when the peak occurs) was 100% for BPA-BNCT, while for Comb-BNCT, Comb-BNCT+O-Fuco and Comb-BNCT+GLN, it was below 70%, with this difference being statistically significant (*p* ≤ 0.05) (Figure 9). Although different percentages of radiodermatitis were observed in the different Comb-BNCT groups, these differences did not reach statistical significance. When we evaluated recovery in the third week, we observed that severe dermatitis persisted in half of the animals treated with Comb-BNCT but only in a quarter or less of the animals treated with Comb-BNCT+O-Fuco and Comb-BNCT+GLN. These findings would indicate an improvement in recovery in the groups treated with O-Fuco and GLN as adjuvants. No dermatitis was observed in the SHAM and BO groups.

The abscopal effect was evaluated in the left non-irradiated leg of the BDIX rats where colon cancer cells were inoculated 2 weeks after T_0_, which is when the BNCT treatment was performed. We analyzed the percentage of animals with tumor volumes below 50 mm^3^ and the mean tumor volume in the left leg (Table 4). We observed a statistically significant difference in the incidence of tumor volumes below 50 mm^3^ between Comb-BNCT and SHAM (*p* ≤ 0.005), and we found a marginally significant difference between Comb-BNCT+O-Fuco vs. SHAM (*p* = 0.0504). Employing this end-point, we found no abscopal effect for the Comb-BNCT+GLN vs. SHAM group. However, when we compared mean tumor volumes, we observed that Comb-BNCT+GLN had a mean tumor volume of 85 mm^3^, half of the value exhibited by the SHAM group, which was 170 mm^3^. This end-point would suggest a possible abscopal effect exerted by Comb-BNCT+GLN.

**Figure 10:**
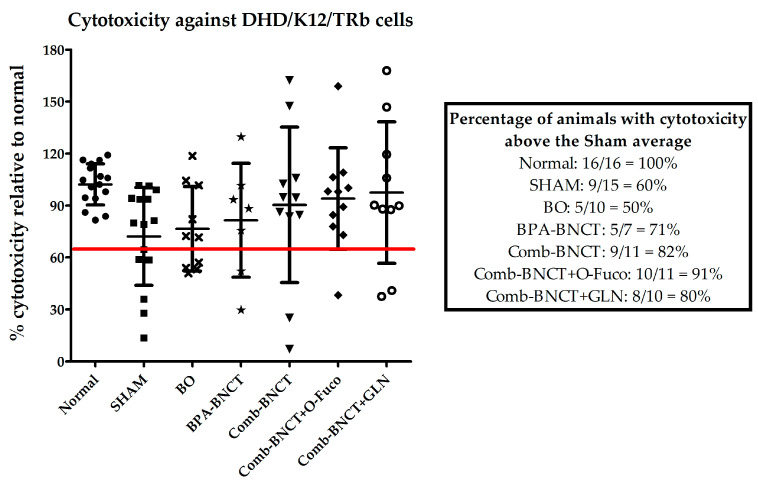
Percentage of systemic cytotoxicity against DHD/K12/TRb relative to normal animals. Comparative table of the percentages of animals with cytotoxicity above the SHAM average.

The percentage of animals with cytotoxicity levels that were higher than the mean of the SHAM group (Figure 10) revealed that the immune response was enhanced for Comb-BNCT vs. BPA-BNCT. In addition, O-Fuco seemed to improve the immune response further.

**Figure 11:**
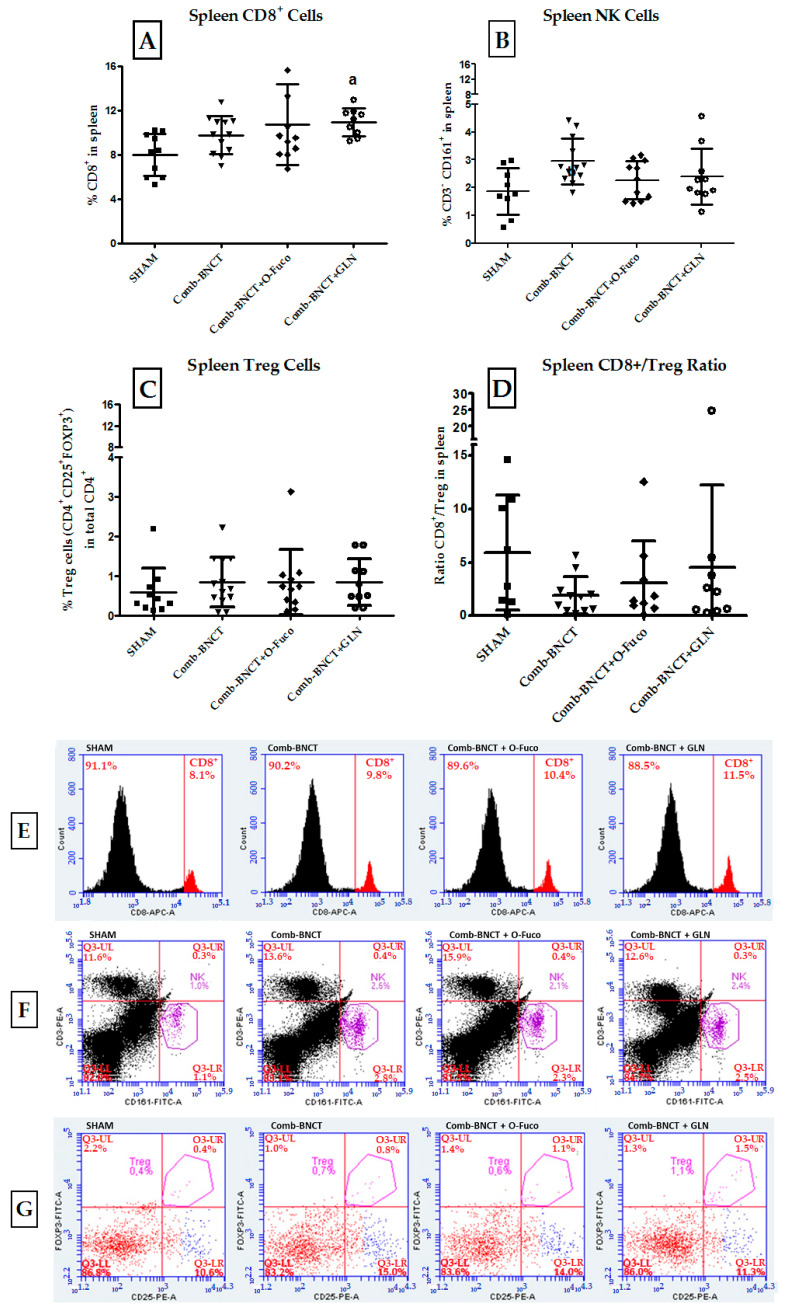
Evaluation of immune cell populations in spleen. (**A**) Percentage of CD8^+^ cells in the spleen, a = *p* ≤ 0.01. (**B**) Percentage of NK cells (CD3^−^CD161^+^) in the spleen. b = *p* ≤ 0.05 vs. SHAM. (**C**) Percentage of Treg cells (CD4^+^CD25^+^FOXP3^+^) in the spleen. (**D**) CD8/Treg ratio in spleen. Representative flow cytometry graphs of splenocytes. (**E**) Histograms show the percentage of CD8^+^ cells in total splenocytes. (**F**) Dot plots show NK cells (CD161^+^CD3^−^) in total splenocytes. (**G**) Dot plots show Treg cells (CD4^+^CD25^+^FOXP3^+^) in total CD4^+^ from spleen.

By analyzing the immune profile at the systemic level, we found a significant increase in the percentage of CD8^+^ cells in Comb-BNCT+GLN compared to SHAM *p* ≤ 0.01. Similarly, NK cells were increased in Comb-BNCT compared to SHAM *p* ≤ 0.05 (Figure 11).

Figure 11A shows that at the level of the systemic immune response, there was a statistically significant increase in CD8^+^ cells in the spleen for the Comb-BNCT+GLN group vs. the SHAM group (*p* ≤ 0.01). Although the Comb-BNCT and Comb-BNCT+O-Fuco groups presented a higher percentage of CD8^+^ cells than the SHAM group, this difference did not reach statistical significance.

As seen in Figure 11B, at the level of the systemic immune response, an increase in NK cells (CD3^−^CD161^+^) was observed for Comb-BNCT vs. SHAM (*p* ≤ 0.05). While for the Comb-BNCT+O-Fuco and Comb-BNCT+GLN groups, there were no significant differences compared to the SHAM group, both groups presented a mean that is above the mean of the SHAM group. We did not find differences in splenic Treg cells between experimental groups.

To analyze the effective immune profile, we assessed the CD8^+^/Treg ratio for each group (Figure 11D). We found no significant differences in the CD8^+^/Treg ratio between the groups analyzed.

## 4. Discussion

This study shows the therapeutic efficacy of BNCT in an ectopic colon cancer model in rats using two boron compounds approved for use in humans. By evaluating the local effect on the growth of the tumors, as a post/pre ratio of the tumor volume in the right leg, we were able to observe a marked reduction in Comb-BNCT (0.7 ± 0.8), where, after 7 weeks of follow-up, not only did the tumor not grow, but it underwent a reduction in tumor volume from its initial volume at the time of irradiation. We observed the same in the protocols where Comb-BNCT was combined with O-Fuco and GLN, indicating that the administration of these compounds did not affect the therapeutic efficacy of BNCT. In contrast, in the SHAM group, tumors grew to more than 10 times their initial volume. The difference in tumor volume at the end of follow-up for the SHAM group and the three Comb-BNCT groups was highly statistically significant (*p* < 0.0001). The parameter “incidence of tumors that underwent a reduction to ≤50% of the initial tumor volume” revealed that more than half of the animals treated with Comb-BNCT underwent a reduction in the tumor volume to 50% or less of the initial volume at the end of follow-up. The incidence of tumors whose volume was reduced to 50% or less of the initial volume was even higher in the groups treated with O-Fuco and GLN as adjuvants. This may be related to previous findings that the compound O-Fuco has anticancer properties [7] and that GLN inhibited the development of tumors in an experimental model of oral cancer [10]. In principle, it would have been contributory to study the effect of Glutamine and Oligo-Fucoidan alone in our model. However, pilot studies in the experimental model studied herein showed that this effect was small or negligible. Within this context, the incorporation of these groups would have implied the use of a greater number of animals with little potential gain, in violation of the 3Rs principle.

Comparing the Comb-BNCT protocol with the standard BPA-BNCT protocol [14], in the same model, we observed that Comb-BNCT (post/pre ratio of tumor volume 0.7 ± 0.8) improved the therapeutic efficacy achieved with the standard BPA-BNCT protocol (post/pre ratio of tumor volume 1.8 ± 1.6) and reduced the severity and duration of dermatitis. The therapeutic efficacy of BPA-BNCT in the BDIX rat ectopic model of colon carcinoma is limited by (1) the solubility of BPA, which, in turn, determines the maximum injection volume, (2) the dose-limiting radiotoxicity (dermatitis) favored due to the preferential uptake of BPA in the basal layer of the skin and (3) the heterogeneous microdistribution of boron in tumor cells because uptake is associated with metabolic activity [14]. Based on previous findings from our group, we tested BNCT mediated by the combined administration of BPA and GB-10, seeking to overcome these limitations in a model of oral cancer [20,21]. GB-10 is a diffusive boron compound that is readily soluble in water, making it easy to implement the administration protocol. It is not selectively taken up by tumor cells. However, as previously described, the selective effect of GB-10-BNCT on the tumor is based on a selective effect on aberrant radiosensitive tumor blood vessels rather than on the selective uptake of GB-10 in the tumor [6]. Tumor boron concentration values for BPA plus GB-10 were higher than for BPA (given at the highest dose/injection volume tolerated by the animals). Although BPA plus GB-10 was not selectively taken up by the tumor compared to exposed skin and blood, the homogeneous distribution of boron would improve therapeutic efficacy. In previous biodistribution studies, using the same combination of boronated compounds as in the present study, we observed that much boron accumulates in the kidney, perhaps due to kidney clearance, and the rest of the organs had values just below the value of the tumor [16]. Therefore, in orthotopic studies, special care should be taken to protect the dose-limiting organs by employing shielding and/or adequate treatment planning. In our study, since the tumor was ectopic and what was exposed to the treatment was the leg that bears the tumor while the rest of the body was shielded from the thermal neutron flux, no adverse effects were observed in the organs. The fact that GB-10 microdistribution studies showed that this compound accumulates preferentially in the tumor stroma would explain a lower incidence of dermatitis [5]. In contrast, BPA preferentially accumulates in the tumor parenchyma, favoring tumor control, but it also preferentially accumulates in the basal layer of the skin, favoring the development of dermatitis. The BPA+GB-10 protocol would combine the advantages of both compounds, minimizing their limitations.

The Boron concentration in the tumor is greater than 20 ppm, considered potentially therapeutic in previous studies [16]. However, a similar concentration was found in the skin surrounding the tumor, which was, in turn, much higher than that in the distal skin. This may be due to infiltrating tumor cells or the fact that the tumor microenvironment gives rise to the neovascularization of the area near the tumor, inducing a greater incorporation of boron into normal tissues. Tumor blood vessels resulting from angiogenesis and vasculogenesis are functionally and structurally altered. They are tortuous and dilated; their walls present fenestrae, vesicles, and transcellular orifices; there are widened interendothelial junctions; and there is absence or discontinuity of the basement membrane [22,23]. Similar features were found in the ARO cell model of undifferentiated thyroid carcinoma (UTC) in NIH nude mice [24].

The use of O-Fuco and GLN as adjuncts to Comb-BNCT proved favorable in terms of shortening the duration of severe dermatitis, probably due to their ability to enhance wound healing and their anti-inflammatory effects [25,26]. Furthermore, a systemic immune response was activated after Comb-BNCT, and O-Fuco appeared to enhance the immune response achieved by Comb-BNCT. Previous in vitro studies showed that O-Fuco supports immune surveillance by affecting the polarization and invasiveness of M2 macrophages (tumor-promoting cells) and repolarizes macrophages toward the M1-like phenotype, which induces antitumor inflammatory reactions [27]. Supplementing olaparib therapy with O-Fuco reprogrammed the plasticity of innate and adaptive immune cells, potentially reprogramming the microenvironment against the tumor [7]. In this sense, future studies will evaluate in more detail the possible immunological mechanisms underlying Comb-BNCT+O-Fuco combination therapy in the BDIX rat ectopic model of colon cancer.

We studied the metastatic spread to the lymph nodes that drain the tumor. This end-point would combine both effects, a local effect due to the nodes that are within the treatment volume and an abscopal effect, since it is the first site where the immune system would be acting. Regional therapeutic efficacy in terms of a statistically significant reduction in metastatic spread was achieved with all Comb-BNCT protocols versus SHAM. Furthermore, we observed a more marked effect when the Comb-BNCT+O-Fuco and Comb-BNCT+GLN protocols were applied. In a breast cancer model, it was observed that Fucoidan can prevent metastasis and proliferation [28]. O-Fuco plus olaparib was shown to efficiently increase cytotoxic T cells in the lymphoid system and more effectively reduce the infiltration of immunosuppressive M2 macrophages and Tregs into the tumor [7]. Additionally, in the same rat ectopic model of colon cancer, we were able to achieve a statistically significant reduction in lymph node metastatic spread when we combined BPA-BNCT and BCG as immunotherapy [14].

Since the abscopal effect is mediated by the immune system, we decided to evaluate the systemic immune profile present in the spleen of the tumor-bearing animals of each experimental group. We observed a superior cytotoxic systemic immune response in all groups treated with Comb-BNCT compared to SHAM. This could indicate that BNCT therapy could activate the immune system to attack distant tumor cells. Likewise, the treatment-induced immune response, in terms of the cytotoxicity assay, was improved for the Comb-BNCT protocol compared to the BPA-BNCT protocol. This effect may be associated with a more robust local tumor response in the case of Comb-BNCT vs. BPA-BNCT as described.

It is known that local irradiation induces a release of mitotic inhibitors (cytokines such as tumor necrosis factor α) into the circulation that mediate a systemic antitumor effect [29,30]. Furthermore, irradiation of the tumor at one site induces the release of circulating tumor antigens, alarmins and inflammatory factors that may then mediate an enhanced immune response against non-irradiated malignancies expressing similar tumor antigens [31,32]. It was shown that the application of BPA-BNCT produces significant local tumor control and is also capable of inducing an abscopal effect through an antitumor response at a distant site [13]. We were able to verify this same effect in this study with a new treatment protocol, using two boron compounds with different incorporation characteristics such as BPA and GB-10 and adjuvants such as O-Fuco and GLN. Although an abscopal effect has been described for neutrons alone [14], the neutron fluence employed herein was not enough to induce an abscopal effect in the Beam only group. We were not only able to observe a local and abscopal effect but also a modulation of systemic immune populations. We observed that the immune cytotoxicity measured for the treatments that included BNCT was higher than for the SHAM group. Likewise, the analysis of the percentage of immune cells suggests that the cytotoxicity in the Comb-BNCT group is mainly mediated by NK cells, while in the Comb-BNCT+O-Fuco or the Comb-BNCT+GLN groups, it would be mediated by CD8^+^. The immunostimulatory effects of local radiotherapy have been shown to be weakened by the microenvironment of established tumors, which is often highly immunosuppressive. Abnormal tumor angiogenesis plays an important role in tumor-induced immune dysregulation [13]. The Treg cells also play an important role in inhibiting tumor rejection by secretion of immunosuppressive cytokines and/or by direct contact with effector T cells [33]. The higher the CD8+/Treg ratio, the better the immune response (although this concept applies much more to the tumor infiltrate and not so much to a systemic response like the one we evaluated in the spleen). In this work, we could not find differences between the groups with this parameter. The Tregs and the CD8+/Treg ratio could be better assessed if a tumor infiltration technique were employed.

These findings are potentially useful in the modern accelerator-based BNCT, where the neutron beams have an epithermal spectrum, designed to penetrate the tissues to effectively treat deep-seated tumors. In some treatments with epithermal beams, skin is the dose-limiting organ due to the enhanced BPA uptake but also because it absorbs the dose due to the scattering of fast neutrons in hydrogen [34,35]. Lowering the skin radiotoxicity, and thus increasing radiotolerance, could play a role in the optimization of the administration of a therapeutic dose to the tumor.

## 5. Conclusions

Previous studies carried out by the research group, added to those derived from this work, continue to confirm that boron neutron capture therapy (BNCT), using nuclear reactors as a neutron source, is effective for the treatment of tumors. Here, we demonstrate that BNCT mediated by the combined administration of two boron compounds such as BPA and GB-10 generates a good antitumor response. Likewise, this strategy improves the therapeutic efficacy achieved with the standard BPA-BNCT protocol and reduces the severity and duration of dermatitis. This response is also associated with the induction of a systemic cytotoxic immune response capable of mounting an abscopal effect that can inhibit the growth of other “out-of-field” tumors. Likewise, the inhibition of metastatic spread in the lymph nodes draining the tumor was observed. We also observed that the addition of Oligo-Fucoidan or Glutamine to the Comb-BNCT treatment does not inhibit its antitumor action but rather maintains it or even improves it, while it exhibits a tendency to decrease dermatitis, one of the major adverse effects of radiation therapies.

## Figures and Tables

**Table 1:** Absorbed Dose (Gy) expressed as mean (min; max) and boron concentration as indicated.

	Leg (Surrounding Skin)	Tumor
**Induced protons (N-14)**	1.0 (0.9; 1.0)	1.0 (0.9; 1.0)
**Total γ ray dose**	1.1 (0.9; 1.3)	1.1 (0.9; 1.3)
**Boron dose**	9.3 (8.4; 9.6)	9.4 (8.7; 9.8)
**ppm ^10^B mean ± SD**	30.7 ± 8.2	31.4 ± 7.1
**Total BNCT dose**	11.4 (10.2; 11.9)	11.5 (10.5; 12.1)

**Table 2:** Boron concentration and ratio in different tissues, mean ± standard deviation (n).

Tissues	BPA 31 mg ^10^B/kg bw + GB-10 34 mg ^10^B/kg bw; iv (ppm) Used for Comb-BNCT	BPA 46.5 mg^10^B/Kg bw; iv (ppm) Used for BPA-BNCT
**Tumor (T)**	31 ± 7 (11)	27 ± 9 (14)
**Surrounding Skin (SS)**	31 ± 8 (12)	23 ± 6 (14)
**Distal skin (DS)**	13 ± 13 (6)	15 ± 2 (6)
**Muscle**	25 ± 6 (12)	25 ± 6 (9)
**Lymph Node**	20 ± 1 (4)	-
**Lung**	24 ± 4 (6)	17 ± 7 (6)
**Blood (B)**	32 ± 7 (7)	14 ± 4 (9)
**T/SS Ratio**	1 ± 0.1	1.2 ± 0.3
**T/DS Ratio**	1.5 ± 0.5	2 ± 0.4
**T/B Ratio**	1 ± 0.3	2 ± 0.5

n = Number of samples.

**Table 3:** Number and percentage of lymph nodes with (+) and without (-) metastases.

Groups (n)	Lymph Nodes with Metastases (+) and %	Lymph Nodes without Metastases (-) and %
**SHAM (16)**	**(14) 87.5%**	**(2) 12.5%**
**BO** [14] **(10)**	**(9) 100%**	**(0) 0%**
**BPA-BNCT** [14] **(9)**	**(6) 67%**	**(3) 33%**
**Comb-BNCT (9)**	**(2) 22%**	**(7) 78%**
**Comb-BNCT+O-Fuco (11)**	**(1) 9%**	**(10) 91%**
**Comb-BNCT+GLN (8)**	**(1) 12.5%**	**(7) 87.5%**

n = Number of lymph nodes.

**Table 4:** Tumor volume in the left leg expressed as the mean ± SD. Number and percentage of animals with tumor volume < 50 mm^3^ at the end of follow-up. n: number of animals.

Groups (n)	Tumor Volume (mm^3^) Mean ± SD	Number and Percentage of Animals with Tumor Volume < 50 mm^3^
**SHAM (30)**	170 ± 188	9 (30%)
**BO [14]** **(10)**	260 ± 157	1 (10%)
**BPA-BNCT [14]** **(20)**	261 ± 205	3 (15%)
**Comb-BNCT (13)**	37 ± 67	10 (77%) ab
**Comb-BNCT+O-Fuco (11)**	58 ± 89	7 (64%) ab
**Comb-BNCT+GLN (10)**	85 ± 75	4 (36%)

a = *p* ≤ 0.05 vs. SHAM, b = *p* ≤ 0.005 vs. BPA-BNCT.

## Data Availability

Not applicable.

## Abbreviations

BCG	Bacille Calmette-Guérin
BNCT	Boron Neutron Capture Therapy
BPA	Borophenylalanine
BSH	Sodium borocaptate
CICUAL	Commission Animal Care and Use Committee
CNEA	National Atomic Energy Commission
GB-10	Decahydrodecaborate
GLN	Glutamine
ICP-OES	Inductively Coupled Plasma Optical Emission Spectrometry
ITVRed ≤ 50%	Incidence of Tumors that underwent a Reduction ≤ to 50% of initial tumor volume
O-Fuco	Oligo-Fucoidan
SPND	Self-Powered Neutron Detector
UTC	Undifferentiated Thyroid Carcinoma
